# Design, Fabrication and Analysis of Magnetorheological Soft Gripper

**DOI:** 10.3390/s22072757

**Published:** 2022-04-02

**Authors:** Jakub Bernat, Piotr Gajewski, Rafał Kapela, Agnieszka Marcinkowska, Paulina Superczyńska

**Affiliations:** 1 Institute of Automatic Control and Robotics, Poznan University of Technology, 60-965 Poznan, Poland; rafal.kapela@put.poznan.pl (R.K.); paulina.superczynska@put.poznan.pl (P.S.); 2 Institute of Chemical Technology and Engineering, Poznan University of Technology, 60-965 Poznan, Poland; piotr.gajewski@put.poznan.pl (P.G.); agnieszka.marcinkowska@put.poznan.pl (A.M.)

**Keywords:** magnetorheological elastomers, MRE, soft gripper

## Abstract

The magnetorheological elastomer is promising material for applications in soft robotics. Its properties like reactive to external magnetic field and softness allow to construct an attractive devices. This work presents a construction of soft gripper assembled with magnetorheological elastomers. The work describes the detailed molding process of magnetorheological elastomers. Further, the electromechanical properties of magnetorheological elastomers are shown using a simple beam. Finally, the soft gripper is constructed and analyzed with the series of experiments.

## 1. Introduction

The development of an intelligent materials gained the focus of researchers and practitioners from various disciplines [1]. The interesting features like softness which is not available in traditional materials like steel causes the possibilities to construct devices easier to operate with delicate objects or to cooperate with humans [2]. This is especially visible in the development of soft pneumatic devices based on the silicone and pneumatic actuation [3]. Alternative to pneumatic actuation can be an electrical or electromagnet stimuli. The first one is for instance exploited in the electroactive polymers to create deformation [1,4]. The second one is popular for instance in the ferroelectric polymers [1], magnetorheological fluids [5,6] and magnetorheological elastomers [7].

In this work, we are focused on the the magnetorheological elastomers (MRE) which are composite materials reinforced by ferromagnetic particles with ferromagnetic and visco-elastic properties [8,9,10]. They are solid analog of magnetorheological fluids with a rubber material instead of a liquid. Different medium does not allow particles to settle with time, increases the flexibility (because the container is not needed) and the permeability (needed to generate larger displacement force). Doping with those particles causes sensitivity to the magnetic fields. Due to the fact that MRE depends on magnetic field it is possible to affect on material by changing magnetic field properties such as amplitude or direction of propagation. Several computational and experimental studies have been carried out in the past of materials with MRE core. These studies were mainly concerned with vibration, damping and responses, and visco-elastic behavior [8,9,10].

In the recent time, the intelligent materials were applied to build various devices for soft robotics [2,11,12]. One of the most challenging tasks is to build soft gripper allows the handling sensitive objects trying to imitate the solutions from nature [2,13]. The soft grippers can be exploited in many area of industrial and domestic applications for instance in medical applications [14] or plant harvesting [15]. The soft grippers can be dived into groups based on the method of actuation such as pneumatic, hydraulic, magnetic field or electric field [2,13]. One of the most interesting method of building soft grippers is application of soft fluid actuators [16] which can be driven by pneumatic or hydraulic excitation. In recent times, these soft grippers has been extensively developed and had a commercial application like PneuNets [17]. Pneumatic soft gripper are build with material possessing the possibility of high stretching and are driven by varying pressure. They enable us to successfully grasp a variety of objects but require compressed air preparation unit to work [2,17]. Beside the success of the soft fluid actuators, the different types of actuation are also used. For instance, in work [18], the Ionic Polymer Metal Composite (IPMC) was applied to build a EMG controlled micro gripper. Another possibility is the Dielectric Electroactive Polymers which was applied to build a gripped driven by high electric field [19]. In the work [20,21] the magnetic soft actuators are summarized to build the milli-, micro- and nanoscale soft devices. The soft magnetic grippers are developed with application of soft permanent magnets and magnetorheologicial fluids [22,23,24].

In this work, the magnetorheological elastomers are applied to build a soft gripper activated by external electromagnet field. Firstly, to show the properties of magnetorheological elastomers, the sample of beam with various content of iron particles are prepared. Then, the series of experiments are conducted which expose the features of MRE soft beam and its reaction to external stimuli. Secondly, the soft gripper performed with MRE material is designed. Its unique construction with electromagnets allow to grasp varying objects. Further, its features using image processing techniques are analyzed.

## 2. Magnetoreohogical Materials

### 2.1. Preparation of Magnetorheological Elastomers

**Figure 1:**
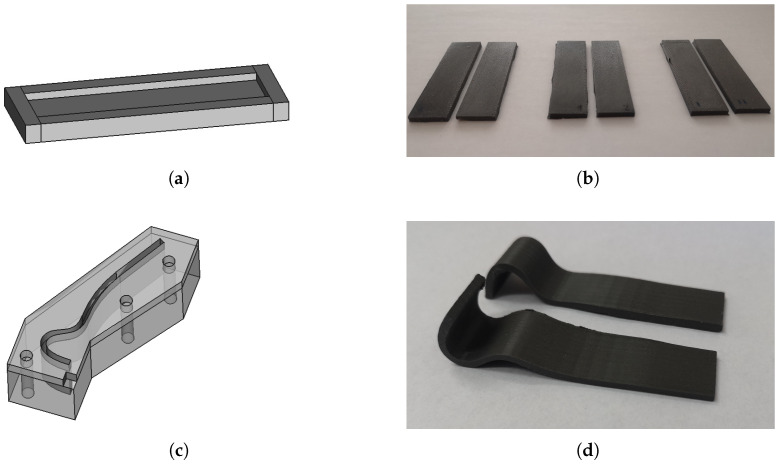
The mold for preparing sample beams (**a**). Example of performed beams (**b**). The gripper finger mold (**c**). The example of gripper finger (**d**).

The magnetorheological elastomers were prepared in the following way: an appropriate amount of RTV-2 silicone (OTT-S825 from OTTSilicone) was weighed into a glass vessel, and then an appropriate amount of an iron powder with an average particle size of 63 μm was added. The ingredients were thoroughly mixed to obtain a homogeneous mixture. Then the OTT-S825 catalyst was introduced in the amount of 2% by weight in relation to the amount of silicone used. After thorough mixing, the prepared system was degassed by vacuum treatment to remove any air bubbles introduced into the mixture during stirring the ingredients. This allowed avoiding foaming of the finished composite MRE sample. Then, the mixture was poured into the prepared molds of beam and gripper finger (Figure 1) and allowed to harden by crosslinking reaction for another 12 h. The MRE composites with three different concentrations: 30%, 50% and 70% by weight of the filler were prepared. As a supplementary material, a document is included containing all dimensions of the gripper finger as well as information about the shape.

### 2.2. Permeability Estimation

The crucial property of the magnetorheological elastomers from electromagnetic point of view is the permeability. It describes the reaction of magnetization on the magnetic field. The higher value of permeability provides that the material stores more magnetic energy or it is more reactive with external magnetic sources.

In this section, we show a simple and fast method of permeability analysis which is suitable for magnetorheological elastomers. It is based on the inductance measurement and it allows to measure the permeability of different MRE beams quickly.

**Figure 2:**
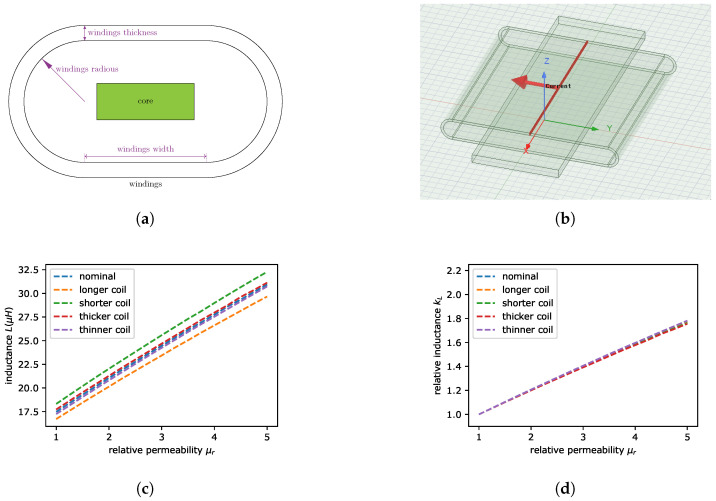
The cross section of coil windings and beam (core) (**a**). The coil model. (**b**) The absolute value of inductance for coil with varying permeability of beam and various geometry (**c**). The relative value of inductance for analogous example (**d**).

Firstly, we will show the idea of measurement in Ansys software (Ansys^®^ Academic Research Electronics 2021 R2) where the model of coil was analyzed for MRE beam with varying permeability as a core. The model used in the simulation is visible in Figure 2a and the cross section is shown in Figure 2b. The dimension are defined in Table 1. In the simulation, we have assumed that MRE permeability is linear and there is no hysteresis effect. The sensitivity of inductance was checked for different dimensions of coil. We have checked the influence of longer, shorter, thicker and thinner coil’s winding. The list coil’s dimensions is given in Table 2. The Ansys software was applied to find the self inductance for all cases. In Figure 2c it is visible that the self inductance is very sensitive to the change of windings dimensions. Therefore, the imperfections for instance in producing the coil, can have strong influence on relative permeability estimation. However, the solution of this problem is to calculate the relative inductance

(1) $$k_{L}\left(\mu_{r}\right)=\frac{L(\mu_{r})}{L(\mu_{0})}$$

where *L* is the inductance which depends on the permeability of core material and $\mu_{0}$ is the relative permeability of coil without core (the relative permeability of vacuum $\mu_{0}=1$). It is visible in Figure 2d that the varying geometry influences the relative inductance much less than absolute inductance presented in Figure 2c.

**Figure 3:**
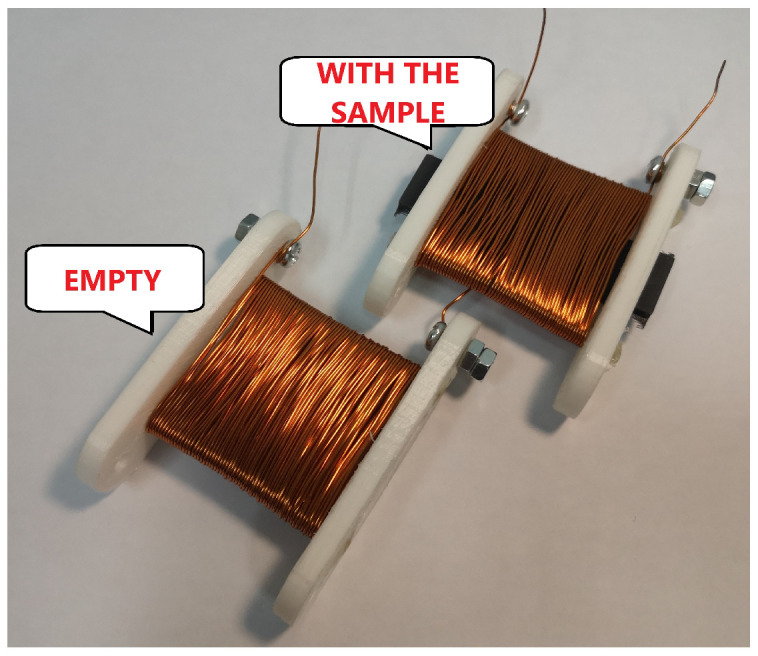
The example of coil built to perform estimate of relative permeability.

To estimate the permeability of magnetorheological elastomers, the two coils were build (one of them is presented in Figure 3). Next, the inductance of MRE beams with various iron powder content was measured by accurate LCR meter (Sanwa LCR700). Based on the measured inductance and characteristics presented in Figure 2d, the permeability of magnetorheological elastomer is estimated by calculating:

(2) $${\hat{\mu }}_{r}=k_{L}^{-1}\left(\frac{L_{s}}{L_{0}}\right)$$

where ${\hat{\mu }}_{r}$ is the estimated magnetic permeability, $k_{L}^{-1}$ is the inverse of relationship between relative inductance and permeability, $L_{s}$ is the inductance of coil with MRE beam and $L_{0}$ is the inductance of coil without core. The results are presented in Table 3. The measurement of relative inductance is much less disturbed by the coil type than the absolute inductance. Further, the permeability of magnetorheological elastomer is low (for instance in compare to electromagnetic steel). It is worth to point that similar range of permeability for MRE is also found in the work [7].

### 2.3. Damping Properties

**Figure 4:**
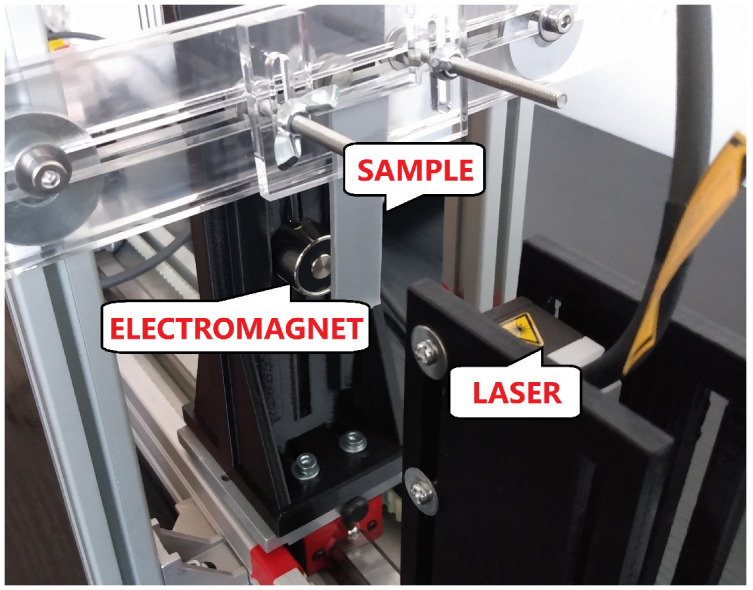
The laboratory set up for analysing the free oscillations of MRE beam.

**Figure 5:**
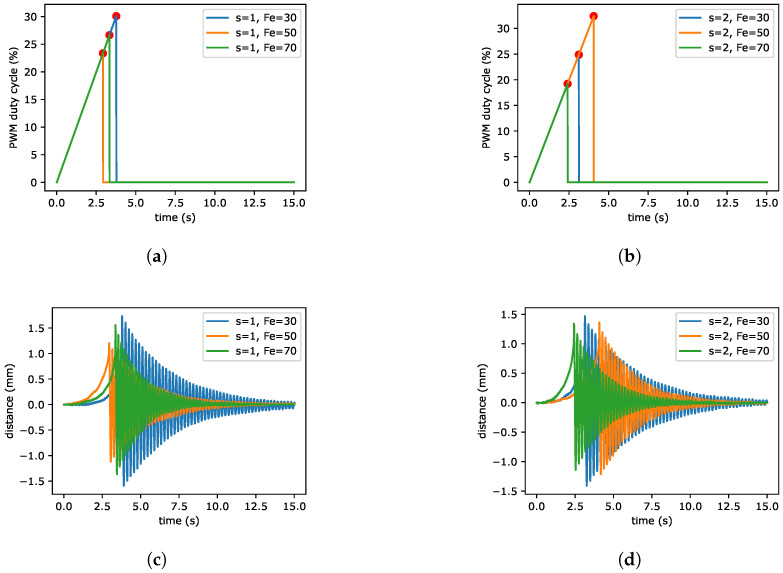
The free vibrations of MRE beam. The duty cycle of PWM and distance response for sample 1 (**a**,**c**). The duty cycle of PWM and distance response for sample 2 (**b**,**d**).

In the case of magnetorheological elastomers, the mechanical properties are also very important. As an example of the mechanical behaviour of MRE, the free damped oscillations of MRE beams were analyzed by performing series of experiments. The laboratory kit was assembled as shown in Figure 4 and its main parts are as follows: MRE beam, electromagnet and laser as distance sensor. In the first step, the electromagnet was activated with linearly increasing current and hence it was pulling the beam. This step is visible in Figure 5 between 0 and around 3 s. The second step is active, if the beam was moved by 1 mm, the electromagnet current was set to 0. Because the pulling force stopped working on the beam, it returns to initial position by a free oscillatory movement. The beam come back to initial position by long damped oscillations. Taking into account the described procedures, all samples with various content of iron powder was analyzed (two samples of each content of iron powder, i.e., 30%, 50% and 70% by weight giving a total number of 6 samples). The example result obtained in the experiments are shown in Figure 5. The input signal expressed as PWM duty cycle is presented in Figure 5a,b and the movement of beam measured by laser is visible in Figure 5c,d.

**Figure 6:**
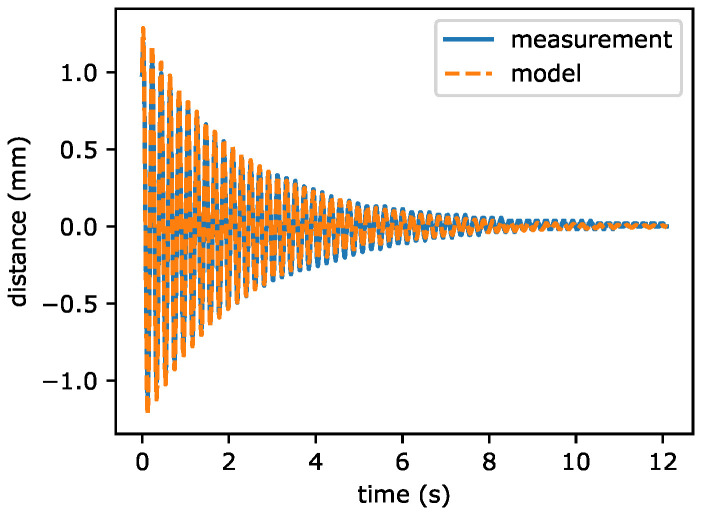
The example of model and measurement transients.

To analyze the performed experiments, the following model of beam response is considered:

(3) $$y\left(t\right)=Ae^{-\alpha t}sin\left(\omega t\right)$$

where *A* is the amplitude of oscillations, α is the decay rate and ω is the angular frequency. The above equation represents a response of second order system. To find the values of *A*, α and ω, the optimization process is carried out. The goal function is defined as follows:

(4) $$J\left(A,\alpha ,\omega \right)=\frac{1}{N}\sum_{k=0}^{N}{\left[y_{m}\left(t_{k}\right)-y\left(t_{k}\right)\right]}^{2}$$

where $y_{m}\left(t_{k}\right)$ is the measured value at time $t_{k}$, $y(t_{k})$ is the estimated value found from Equation (3) and *N* is the number of measured points. The optimization was performed by Nelder-Mead algorithm implemented in scipy [25]. The example of estimated output obtained by identification is presented in Figure 6. It is visible that good agreement between the model and measurement is obtained.

Relying on the above procedure, all responses of MRE beam were analyzed. The obtained results are presented in Table 4. It can be seen that the content of iron in silicone matrix has strong influence on the decay rate and settling time (these parameters are correlated). It can be easily stated that increasing content of iron powder in the silicone matrix causes the decreasing value of the settling time and increasing value of the decay rate. It means that the higher content of iron powder in MRE implies more damped responses. Further, the angular frequency is much less sensitive to the varying Fe content.

### 2.4. Magnetic Attraction Force Experiment

**Figure 7:**
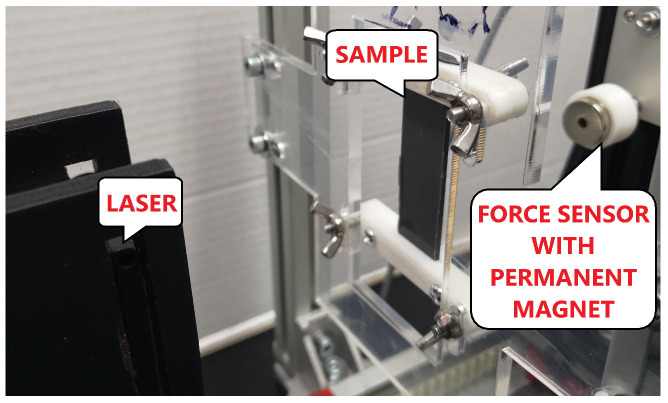
The laboratory set up for analysing the force of MRE beam.

**Figure 8:**
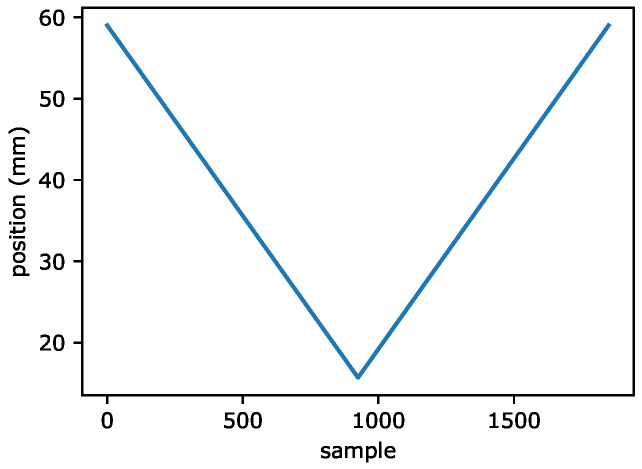
The permanent magnet position in the measurement of force.

**Figure 9:**
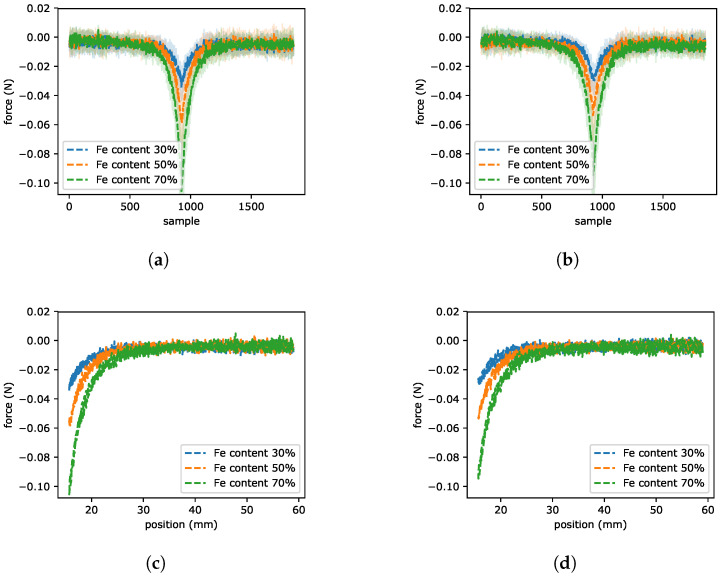
The force measurement between soft beam and permanent magnet. The force sensor for sample 1 (**a**) and sample 2 (**b**). The representation of force as a function of the position for sample 1 (**c**) and sample 2 (**d**).

Experiment presented in this section was to check the difference in the reaction of created beams with different iron content to the magnet approaching them. The laboratory kit was assembled as presented in Figure 7. The main parts are: MRE beam and the permanent magnet placed on the distance sensor. The beam had fastened both ends to avoid any vibrations resulting from free hanging. The permanent magnet with the force sensor approached and moved away from the test object on a distance between 60 and 15 mm as it is illustrated in Figure 8. Experimental results for the magnetic attraction force of the MRE beam are visible in Figure 9. As the magnet moved closer the beam was leaning towards the magnet, what is possible to observe in Figure 9a,b between 750 and 1250 sample. At the point of maximum deflection, and also the strongest force of attraction, the tested element did not touch the magnet, it was tilted to its maximum. As the magnet was receded the MRE returns to its initial position. As in the previous section, all beams samples with various content of Fe powder were analyzed (doubled samples of 30%, 50% and 70% iron powder giving a total number of 6 samples). The example of the input signal expressed as magnet position is presented in Figure 8. Figure 9c,d presents the force as a function of the position for sample 1 and 2, respectively. The maximum force is connected with the maximum content of Fe in the sample. It is around 0.1 N for both tested beams at a distance of around 15 mm.

### 2.5. Tensile Strength Studies

Mechanical properties of magnetorheological elastomers are very important characteristics of material for industrial applications. Thus, to characterize the mechanical properties of the investigated materials the tensile strength was studied. The tests of prepared beams samples were made most of all to estimate the influence of the filler on silicone matrix stiffness. The measurements were performed at universal testing machine Zwick/Roell model Z020 (Zwick GmbH & Co. KG, Ulm, Germany) at 25 ${}^{{}^{\circ}}C$ according to PN-EN ISO 527-1:1998 (crosshead speed of 5 mm min^−1^). Results are presented in Table 5.

The stiffness (Young modulus, $E_{mod}$) of the tested materials increases nonlinearly with the increase of iron content in the composites. This is consistent with the literature [26,27], because inorganic fillers introduced to polymer matrix cause stiffening of the polymer matrix. For materials containing up to 50% by weight, this increase is slight, while 70% by weight of the filler has a greater impact on this parameter—it rises almost four times. Contrary elongation at break decreases with increasing content of Fe in the polymer matrix. Tensile strength is not so sensitive on filler content, and slightly decrease for samples containing 30% and 50% by weight of Fe and slightly increase for sample with 70% by weight of Fe is observed.

## 3. Soft Magnetorheological Gripper

In this section, the idea of the gripper from prepared MRE materials is presented. The main features of that kind of device are softness and reaction on the magnetic field. Relying on these attributes the gripper construction was proposed.

### 3.1. Construction

**Figure 10:**
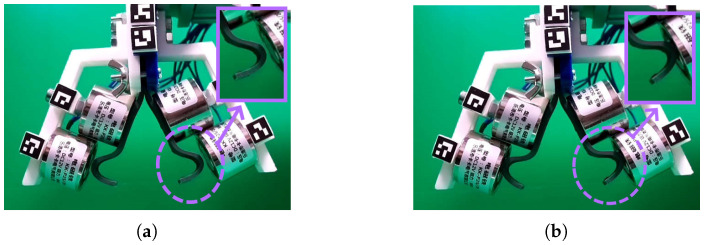
The soft gripper reaction on electromagnet. The upper pair of electromagnets turned on (**a**). The both pairs of electromagnets turned on (**b**).

The construction of gripper is based on two MRE fingers. The shape of single finger is presented in Figure 1c and it was performed based on the form presented in Figure 1d. The curved shape of fingers causes an initial force to be presented between them in the closed position. This force is caused by the elasticity of MRE material. To open the gripper, the electromagnets are installed and activated. The electromagnet creates magnetic field which produce a force acting on MRE finger. The force pulls the fingers to electromagnet and gripper is open. It is worth noting that an important aspect of the work of the MRE fingers is the location of the electromagnets. As shown in Figure 9c,d, the force between the source of the magnetic field and the MRE increases rapidly over short distances. Therefore, in the presented gripper, the position of the electromagnet is crucial. This can be seen in Figure 10 where two configurations of the electromagnet are considered. If the upper electromagnet (which is very close to the finger) is turned on, then MRE is pulled to the electromagnet as shown in Figure 10a. Then, the distance between the lower electromagnets is closer and hence turning on the lower electromagnets causes that fingers are pulled by all electromagnets as is visible in Figure 10b. If only the lower electromagnets are turned on, the force acting on fingers is too low for pulling them, so the fingers stay in the close position.

### 3.2. Experiments

The series of experiments with gripper were performed to show its ability to handling a mass. The set of loads with cylinder shape with different mass were prepared. The steps of experiments were as follows:

- Gripper raised with closed fingers;
- Gripper fingers opened by turning on electromagnet;
- Gripper lowered to load level;
- Gripper fingers closed by turning off electromagnet;
- Gripper raised with mass;
- Gripper fingers opened to release mass.

The procedure was tested for loads with mass: 2.62 g, 2.95 g, 3.28 g, 3.45 g, 3.54 g, 3.62 g, 3.75 g and 3.89 g. In the case of masses 4 g and more, the gripper cannot hold the mass.

#### 3.2.1. Camera System

**Figure 11:**
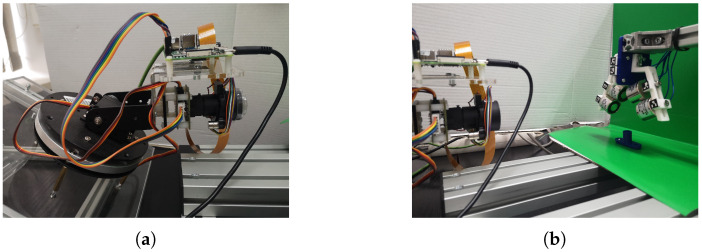
Camera system. Pan–tilt–zoom camera system mount (**a**). The view of the gripper framework (**b**).

Figure 11 shows the camera system implemented for our mass holding experiments. It consists of the pan–tilt–zoom camera system [28], custom camera mount, Raspberry Pi 3 embedded computer and a PC computer. Raspberry computer acts as a central server in this architecture that is responsible for control of the PTZ camera system movement and image capturing. It is also capable of computing of some preprocessing steps like finding AruCo markers [29] or basic light/contrast image enhancements. For image capturing and sharing processes a file server is used. Entire communication takes place by http GET/PUT requests that embed JSON format messages sent after the message header shown below:

{
  "doAF" : 0,
  "y" : 0,
  "x" : 0,
  "focus" : 0,
  "zoom" : 0,
  "imgNo" : 5,
  "imgName" : "test_"
}
		  
where particular fields denote the following functionality:

- doAF—perform autofocus algorithm;
- x,y—set pan/tilt position;
- focus—set focus position;
- zoom—set zoom position;
- imgNo—set experiment number (included in image names in file server).

#### 3.2.2. Gripper Visual Analysis Algorithm

**Figure 12:**
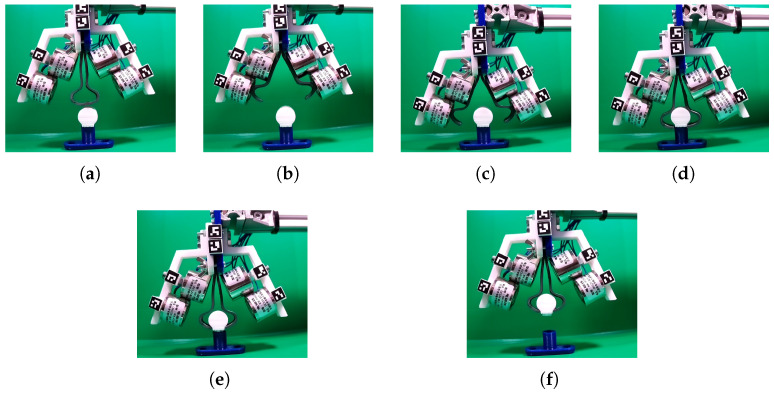
Gripper positions during weight lift experiment. Base position, closed gripper (**a**). Base position, opened gripper (**b**). Catching position, opened gripper (**c**). Catching position, closed gripper (**d**). Initial lifting position, closed gripper (**e**). Goal lifting position, closed gripper (**f**).

The series of photos during experiments include the most significant situations during the gripper performance analysis. These include (Figure 12):

- Closed gripper in the base position (Figure 12a);
- Opened gripper in the base position (Figure 12b);
- Opened gripper in the catching position (Figure 12c);
- Closed gripper in the catching position (Figure 12d);
- Closed gripper in the initial lifting position (Figure 12e);
- Closed gripper in the goal lifting position (Figure 12f).

From the visual analysis point of view, there were two major experiments conducted to calculate gripper characteristics: the maximum gap between the gripper fingers and the flexibility analysis of the displacements under variable load. To achieve that, a custom computer vision algorithm has been built. In order to perform the analysis in a robust way it consists of the preprocessing step where the important features are extracted and the actual analysis of the displacements between two analyzed gripper positions.

**Figure 13:**
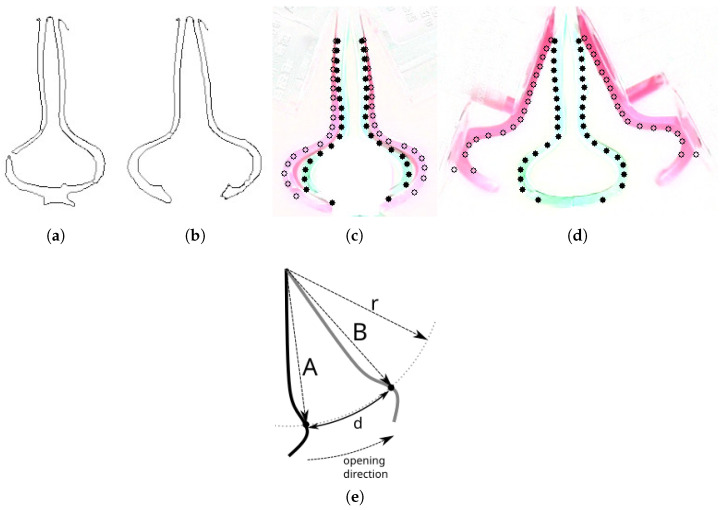
Preprocessing stages of the gripper displacement analysis. Edge image of closed gripper in the base closed position (**a**). Edge image of the closed gripper holding an object (**b**). Difference image between the closed empty gripper and closed gripper holding an object (analysis points marked) (**c**). Difference image between the closed empty gripper and opened gripper (analysis points marked) (**d**). The idea of identification of displacement analysis points (**e**).

By controlling the background color and the lighting conditions (Figure 11) it is easier to perform the preprocessing analysis. In addition, the task is to analyze the displacement of the gripper fingers so assuming the camera position does not change during the experiment all the important features can be extracted from the difference of the two images. Under these assumption it is possible to extract gripper fingers envelope shape for each gripper position knowing the work area and the background color. This is done by detecting the edges in the given image (Figure 13a,b).

We have wanted to perform analysis of the fingers deformations with respect to the distance to the center of the gripper that is located in its mount point. For this reason a number of consecutive points is proposed along the major axis of the gripper (Figure 13c) and (Figure 13d). The way the points locations are extracted is that the algorithm finds a cross-section of the gripper outer envelope and a circle with given radius (*r*) and the center located at the mount point of the gripper. Then the deformation at the given point is measured as a difference between the arc lengths in the two fingers positions accordingly to the Formula (5).

(5) $$d=∥\vec{r}∥\arccos\left(\vec{A}\;\cdot \;\vec{B}\right)$$

#### 3.2.3. Gripper Visual Analysis Algorithm Result Analysis

The major experiment conducted with the gripper was the analysis of weight lifting capabilities. It was performed by the series of single weight lifting experiments with a weight as a parameter. The visual algorithm described in the previous paragraph allowed to measure the deformations along the major axis of the gripper.

In order to measure the maximum opening gap of the gripper the last pair of the envelope points is calculated at the outer edge of the opened gripper (Figure 13d). The arc distance measured by the application was 44 mm and the chord distance was 29 mm calculated using the Formula (6).

(6) $$d=2∥\vec{r}∥\sin\left(0.5\vec{A}\;\cdot \;\vec{B}\right)$$

In the next experiment the flexibility of the gripper was tested. In order to conduct an analysis, the gripper was loaded with variable mass and visual analysis was performed to detect the displacements between the base position and the final position in which gripper holds the weight. Figure 13c,d show the idea behind the analysis. Filled dots are the ones that denote gripper shape envelope in the base position—the empty dots denote gripper envelope in mass holding position. For each *r* there are two pairs of points (left/right) for displacement calculations. Note, that since the fingers of the gripper are rotary the displacement is calculated along the arc of the given *r* (5).

**Figure 14:**
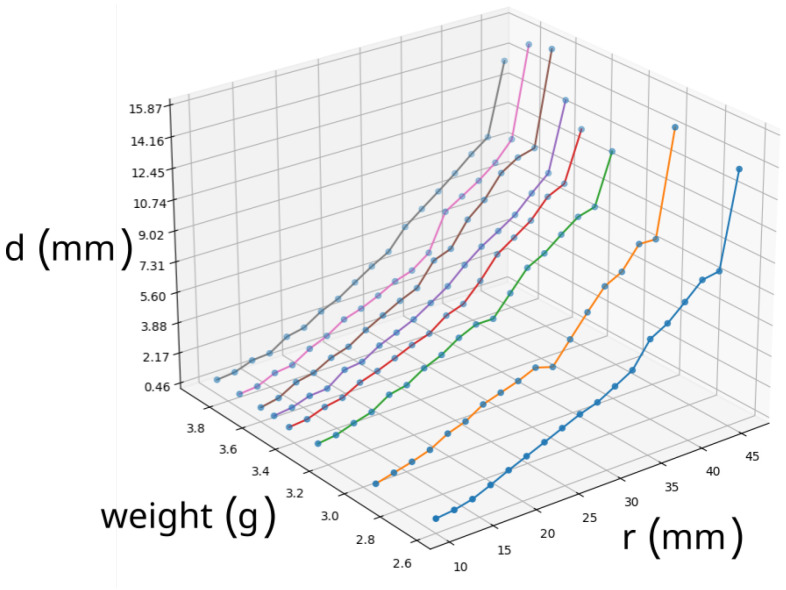
Results from visual analysis. Each line represents the displacement *d* caused by the particular weight along with the radius *r* millimeters from the gripper center.

Figure 14 shows the results of the experiment. The following curves denote the displacements *d* for the same weight along the radius *r*. As it can be seen, there is a tendency that bigger masses introduce bigger displacements since all the curves tend to increase values for heavier masses. In addition the displacement increases for increasing *r* which is obvious for rotary flexible gripper since the flexibility increases over the length of the material. It is also worth to mention that the last displacement for all the weights is always significantly bigger since this is the place where most of the mass is being hold by the gripper.

**Figure 15:**
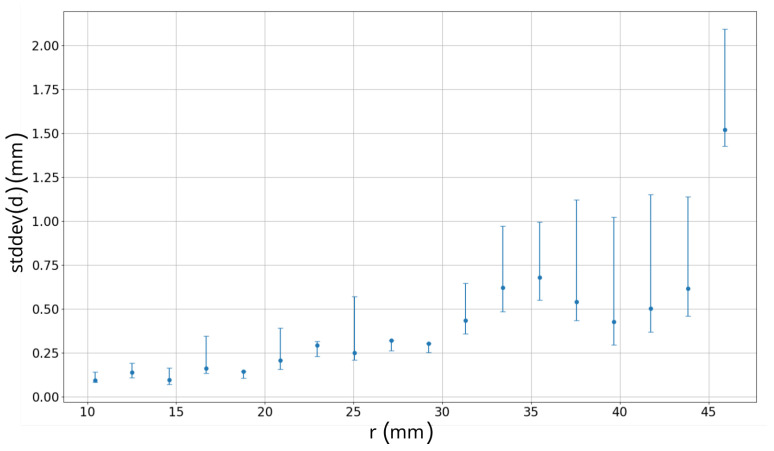
Results from visual analysis. *X* axis represents radius *r*. *Y* axis represents average standard deviation for all the weights with upper and lower error bands.

Figure 15 presents standard deviation of the displacements for each measurement point with respect to the radius. Furthermore, positive and negative error bands are included. As can be observed the measurement point with the longest distance from the gripper center is featured with the highest standard deviation. This is most likely due to the accumulation of the flexibility along the gripper fingers. The incline trend in the rest of the observations also confirms this effect. Another observation made is that for increasing weight the positive error of standard deviation also increases. This might be related to two factors: weight disturbances (uneven weight base position or mass placement insight weight) or bending effect of the gripper fingers which occurs frequently at some specific stress for the fingers. It is also worth to mention that positive error is usually bigger than the negative one since the gripper is stretching by the weight.

### 3.3. Gripping Objects with Varying Shape

To observe possible applications for invented gripper, we have performed gripping test for objects with different shapes, weights, sizes and textures. Table below (Table 6) presents characteristic of chosen objects.

**Figure 16:**
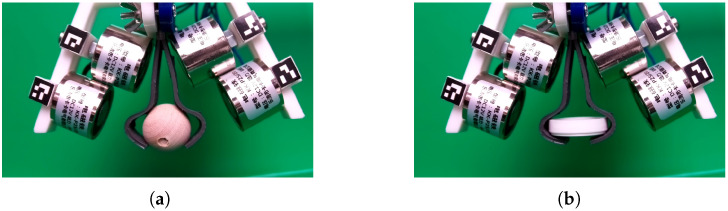

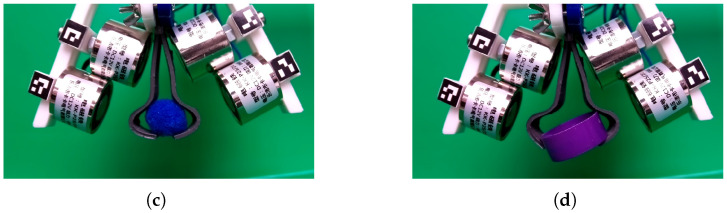
The soft gripper holding various objects: wood ball (**a**), game pawn (**b**), pompon (**c**), pencil sharpener (**d**).

The results of the gripping test are shown in Figure 16. All varying shape objects, were lifted up and did not slip out what proves that invented gripper may be used in different applications and easily grasp desired target. It is also possible to observe that the gripper’s flexibility allows to deflect and fits to gripped object in order to lift it up. The stiffness of the used materials and forces resulting from a specific shape, affect on the object to hold it the during lifting process.

## 4. Conclusions

This article describes a proposal of a soft actuator, presenting its manufacturing process, analysis of permeability and damping properties as well as an experiment of the magnetic attraction force. In the first part of the work, the influence of iron powder content on the properties of MRE was investigated. For this purpose, beams containing 30%, 50% and 70% by weight of iron powder were prepared. On the basis of the performed studies, it was determined that the magnetic properties of investigated samples depend on the iron powder content in MREs. The material with the highest magnetic permeability contains the highest amount of filler (70 wt%) and this MRE was used as a soft actuator—the gripper. In the last section, the operation of the gripper was verified by checking the maximum lifting capacity and deflection of the material during the performed task. In summary, the proposed soft gripper can grasp varying shape objects. The gripper fingers have a moderate possibility to fit external shape. One of the advantages of gripper is the absence of required external stimuli in the closed position. The proposed shape of finger can also be modified to catch different group of objects. In the future, a numerical approach will be used for further evaluation and optimization of the final shape of the MRE actuator.

## Figures and Tables

**Table 1:** The dimensions of beam and coil.

Name	Value
beam length	60 mm
beam width	15 mm
beam thickness	2 mm
winding length *L*	40 mm
winding radius	2 mm
winding thickness *z*	0.6 mm
winding width	36 mm

**Table 2:** The various coil models in the analysis.

Name	Length (*L*)	Thickness (*z*)
nominal	40 mm	0.6 mm
longer	42 mm	0.6 mm
shorter	38 mm	0.6 mm
thinner	40 mm	0.5 mm
thicker	40 mm	0.7 mm

**Table 3:** The results of the inductance measurements for varying iron powder content with the calculated relative inductance and the estimated magnetic permeability.

Coil	Fe Content (%)	L (μH)	$k_{L}\times 100$	${\hat{\mu }}_{r}$
A	-	22.78	1.0	1.0
B	-	25.10	1.0	1.0
A	30	23.52	3.2	1.12
B	30	26.10	4.0	1.16
A	50	25.05	10.0	1.47
B	50	27.58	9.9	1.46
A	70	28.73	26.1	2.31
B	70	31.50	25.5	2.28

**Table 4:** The settling time of free oscillations for samples of beams with different iron content in MRE.

Sample	Fe Content (%)	Settling Time (s)	α (1/s)	ω (rad/s)
1	30	11.2	0.29	28.5
2	30	11.2	0.29	27.6
1	50	8.9	0.45	30.4
2	50	8.0	0.48	31.6
1	70	6.5	0.63	33.9
2	70	6.6	0.56	30.2

**Table 5:** The results of the mechanical strength measurements (Young modulus $E_{mod}$, tensile strength $\sigma_{max}$ and elongation at break $\varepsilon_{max}$) for magnetorheological elastomers with various iron powder content.

Fe Content (%)	$E_{\mathrm{mod}}$ (MPa)	$\sigma_{\max}$ (MPa)	$\varepsilon_{\max}$ (%)
0	0.31±0.029	1.55±0.13	255±19
30	0.42±0.045	1.46±0.15	227±17
50	0.62±0.052	1.43±0.17	179±9.0
70	1.17±0.18	1.71±0.10	103±2.5

**Table 6:** Characteristic of gripped objects.

Dimensions	Wood Ball	Game Pawn	Popmpon	Pencil Sharpener
Weight (g)	2.79	0.8	0.18	2.06
Diameter (mm)	20	25.5	16	26
Height (mm)	-	6.5	-	12

## Supplementary Materials

The following supporting information can be downloaded at: https://www.mdpi.com/article/10.3390/s22072757/s1.
